# Bim expression in endothelial cells and pericytes is essential for regression of the fetal ocular vasculature

**DOI:** 10.1371/journal.pone.0178198

**Published:** 2017-05-26

**Authors:** Shoujian Wang, Ismail S. Zaitoun, Ryan P. Johnson, Nasim Jamali, Zafer Gurel, Catherine M. Wintheiser, Andreas Strasser, Volkhard Lindner, Nader Sheibani, Christine M. Sorenson

**Affiliations:** 1 Department of Ophthalmology and Visual Sciences, University of Wisconsin School of Medicine and Public Health, Madison, Wisconsin, United States of America; 2 Department of Pediatrics, University of Wisconsin School of Medicine and Public Health, Madison, Wisconsin, United States of America; 3 McPherson Eye Research Institute, University of Wisconsin School of Medicine and Public Health, Madison, Wisconsin, United States of America; 4 The Walter and Eliza Hall Institute of Medical Research, University of Melbourne, Melbourne, Victoria, Australia; 5 Department of Medical Biology, University of Melbourne, Melbourne, Victoria, Australia; 6 Center for Molecular Medicine, Maine Medical Center Research Institute, Scarborough, Maine, United States of America; Medical College of Wisconsin, UNITED STATES

## Abstract

Apoptosis plays a central role in developmental and pathological angiogenesis and vessel regression. Bim is a pro-apoptotic Bcl-2 family member that plays a prominent role in both developmental and pathological ocular vessel regression, and neovascularization. Endothelial cells (EC) and pericytes (PC) each play unique roles during vascular development, maintenance and regression. We recently showed that germline deletion of Bim results in persistent hyaloid vasculature, increased retinal vascular density and prevents retinal vessel regression in response to hyperoxia. To determine whether retinal vascular regression is attributable to Bim expression in EC or PC we generated mice carrying a conditional Bim allele (Bim^Flox/Flox^) and VE-cadherin-cre (Bim^EC^ mice) or Pdgfrb-cre (Bim^PC^ mice). Bim^EC^ and Bim^PC^ mice demonstrated attenuated hyaloid vessel regression and postnatal retinal vascular remodeling. We also observed decreased retinal vascular apoptosis and proliferation. Unlike global Bim -/- mice, mice conditionally lacking Bim in EC or PC underwent hyperoxia-mediated vessel obliteration and subsequent retinal neovascularization during oxygen-induced ischemic retinopathy similar to control littermates. Thus, understanding the cell autonomous role Bim plays in the retinal vascular homeostasis will give us new insight into how to modulate pathological retinal neovascularization and vessel regression to preserve vision.

## Introduction

Retinal vascular cell death or apoptosis can be positively or negatively influenced by Bcl-2 family members. Bim is a Bcl-2 homology 3 (BH3) domain-only apoptosis initiating Bcl-2 family member. Unlike other pro-apoptotic Bcl-2 family members with three BH domains, Bim can bind anti-apoptotic family members and promote apoptosis [1]. Studies from our laboratories have shown that Bim not only acts in opposition to Bcl-2 with regards to modulation of apoptosis but also by modulating cell adhesion and migration [2]. We have shown that vascular cells lacking Bim are more adhesive and resistant to apoptotic stimuli while cells lacking Bcl-2 are less adhesive and prone to apoptosis [3]. Lack of Bim or Bcl-2 also resulted in opposing changes in cell migration which were cell type specific [2–4]. Thus, modulating Bim expression may play a crucial role during development and/or remodeling processes including tissue vascularization.

Angiogenesis allows for the expansion of existing blood vessels during development and disease, while remodeling occurs in response to changes in need for oxygen and nutrients. Under normal circumstances, pruning of capillaries in the vascular plexus is followed by stabilization and maturation of the remaining vessels. However, during pathological remodeling in diseases such as retinopathy of prematurity and diabetic retinopathy, vascular remodeling goes awry. The inability to undergo vessel regression following development can also lead to disease such as persistent fetal vasculature. Thus, proper regulation of apoptosis-driven vascular remodeling is essential for development and maintenance of the ocular vasculature.

Although Bim expression is required for endothelial cell apoptosis during VEGF blockade and tumor regression [5], its role during remodeling of the retinal vasculature is not well defined. Our previous studies demonstrated precocious formation of the deep vascular plexus, decreased retinal vascular remodeling, attenuated hyaloid vessel regression and protection from oxygen-induced ischemic retinopathy (OIR) in global Bim deficient (Bim -/-) mice [6]. OIR is characterized by attenuation of retinal vascular development and apoptosis-driven loss of existing blood vessels [7]. This is followed by the ischemic retina initiating a proangiogenic response, including increased VEGF production resulting in pathological growth of new blood vessels. We have previously shown that lack of Bim expression resulted in increased VEGF production in retinal vascular cells [2, 8, 9], which protected the developing retinal vasculature from hyperoxia-mediated vessel obliteration and subsequent neovascularization [6]. Therefore, Bim plays an important role in both normal and aberrant retinal vascular remodeling and neovascularization.

Although apoptosis facilitates ocular vessel regression, the contribution of Bim expression in endothelial cells (EC) and pericytes (PC) during regression and neovascularization is not well understood. EC and PC each play unique roles during development, maintenance and regression of the retinal vasculature. To determine whether the contribution of Bim expression during developmental and pathological vascular regression was attributable to its expression in EC or PC, we generated mice carrying a conditional Bim allele (Bim^Flox/Flox^) and VE-cadherin-cre (Bim^EC^ mice) or Pdgfrb-cre (Bim^PC^ mice). We observed attenuation of hyaloid vessel regression and postnatal vascular pruning in mice lacking Bim in EC or PC. Apoptosis and proliferation were also decreased in the retinal vasculature of Bim^EC^ and Bim^PC^ mice. However, unlike global Bim -/- mice, substantial hyperoxia-mediated vessel obliteration and subsequent neovascularization was observed following OIR in both Bim^EC^ and Bim^PC^ mice [6]. These studies increase our understanding of how modulating apoptosis of specific vascular cells influences not only normal development but also pathological ocular conditions in which vessel regression may be of therapeutic benefit.

## Materials and methods

### Ethics statement

Experiments were performed in accordance to the Association for Research in Vision and Ophthalmology Statement for Use of Animals in Ophthalmic and Vision Research and were approved by the Institutional Animal Care and Use Committee of the University of Wisconsin School of Medicine and Public Health. Euthanasia of animals was done according to approved protocols by CO2 asphyxiation.

### Animals

The VE-cadherin-cre transgenic mouse line (B6.Cg-Tg(Cdh5-cre)7Mlia/J; Jackson Laboratory, Bar Harbor, ME; stock number 006137) genotyping was accomplished by PCR analysis of genomic DNA extracted from tail biopsies using the following primers 5’-GCGGTCTGGCAGTA AAAACTATC-3’ and 5’-GTGAAACAGCATT GCTGTCACTT-3’. The Tg(Pdgfrb-cre)^*45Vli*^ mouse line has been described previously [10] and was screened using the following primers: 5’-GCATTTCTGGGGA TTGCTTA-3’ and 5’-CCCGGCAAAACAGGTAGTTA-3’. The Bim^Conditional^ mice homozygous for this allele were healthy [11]. These mice were screened with the following primers: 5’-AACCAACTGRACCTT GGCTATA-3’ and 5’-GACAAGGTGGACAATTGCAG-3’. These transgenic mice were previously characterized and utilized in numerous studies [10–16].

**Fig 1:**
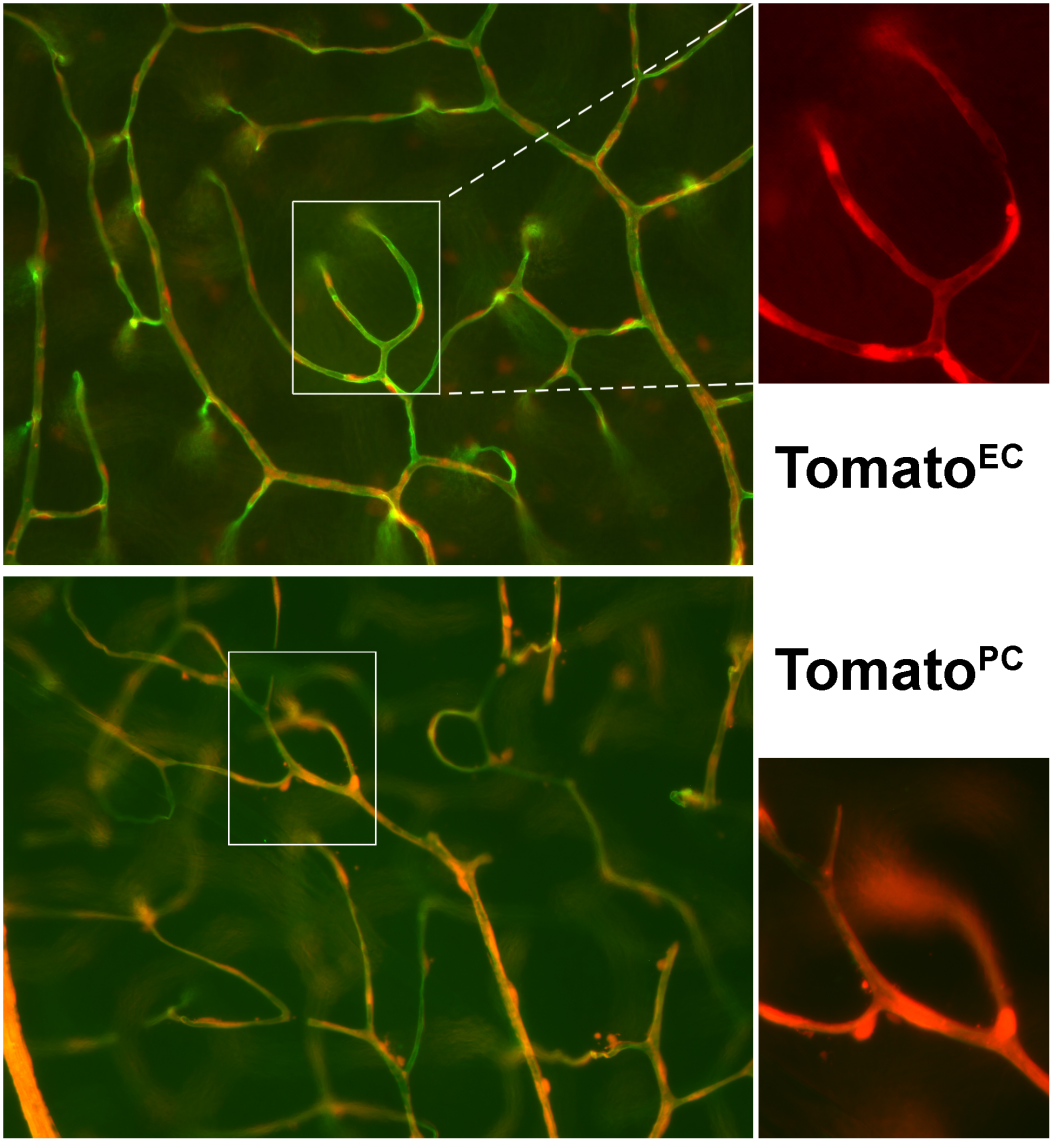
P21 Tomato^EC^ and Tomato^PC^ mice were perfused with FITC-wheat germ agglutinin to visualize the vasculature (green) with VE-cadherin-cre expressing cells (endothelial cells; red) in the upper panel and Pdgfrb-cre expressing cells in the lower panel at x200 with a higher magnification (Tomato) shown at x400.

We crossed mice homozygous for the Bim^Conditional^ allele with either Tg(Pdgfrb-cre)^*45Vli*^ [10] or VE-cadherin-cre (Jackson stock number 006137) mice. The progeny of this cross that were heterozygous for the Bim^Conditional^ allele and expressed either Pdgfrb-cre or VE-cadherin-cre were then crossed. The progeny were screened as described above and mice homozygous for the Bim^Conditional^ allele which also expressed Pdgfrb-cre or VE-cadherin-cre were bred to mice expressing the Bim^Conditional^ allele and genotyped. Mice homozygous for the Bim^Conditional^ allele expressing Pdgfrb-cre are referred to as Bim^PC^ mice. Mice homozygous for the Bim^Conditional^ allele expressing VE-cadherin-cre are referred to as Bim^EC^ mice. C57BL/6J mice, the background strain of the Bim^EC^ or Bim^PC^ mice were used as controls and referred to as wild-type (WT) unless noted otherwise. For example, Bim^Flox/Flox^ littermates were used in OIR studies as noted in the figure legend. To assess Cre-mediated excision we crossed mice carrying a conditional Tomato allele (B6.Cg-Gt (ROSA) 26Sortm14 (CAG-tdTomato)Hze/J; Jackson stock number 007914) to VE-cadherin-cre (Tomato^EC^) or Tg(Pdgfrb-cre)^*45Vli*^ (Tomato^PC^) mice. In some cases, Tomato^EC^ and Tomato^PC^ mice were perfused with FITC-Wheat-germ agglutinin to visualize the vasculature. In the presence of a tissue-specific Cre recombinase, the Tomato cassette was expressed and used to track Cre expression. Tomato expression in the retina was confined to EC in Tomato^EC^ mice and PC in Tomato^PC^ mice (Fig 1). All mice were maintained at the University of Wisconsin animal facilities where our studies were performed according to approved protocols.

For OIR studies, 7-day-old (P7) pups and mothers were exposed to an atmosphere of 75±0.5% oxygen for 5 days. The incubator temperature was maintained at 23±2°C, and oxygen was continuously monitored with a PROOX model 110 oxygen controller (Reming Bioinstruments Co., Redfield, NY). Mice were then brought to room air for up to 5 days, and retinal wholemounts prepared from P17 pups that weighed greater than 6.5 grams as described below.

### Trypsin-digested retinal vessel preparation

Eyes were enucleated from P21 or P42 mice and fixed in 4% paraformaldehyde for at least 24 h. The eyes were bisected equatorially and the entire retina was removed using the dissecting microscope. Retinas were washed overnight in distilled water then incubated in 3% trypsin (Trypsin 1:250, Difco) prepared in 0.1 M Tris, 0.1 M maleic acid, pH7.8 containing 0.2 M NaF for 1–1.5 h at 37°C. Following digestion, the retinal vessels were flattened with four radial cuts, then mounted on glass slides for periodic acid-schiff (PAS) and hematoxylin staining. Nuclear morphology was used to distinguish PC from EC. The nuclei of EC are oval or elongated. They lie within the vessel wall along the axis of the capillary. PC nuclei are small, spherical, stain densely, and usually have a protuberant position on the capillary wall. The stained retinal wholemounts were coded, and subsequent counting was performed masked.

EC and PC numbers were determined by counting respective nuclei per field of view under the microscope at a magnification of x400. Only vascular cells on retinal capillaries were counted, which was performed in the mid-zone of the retina. We counted the number of EC and PC in four fields of view from each of four quadrants of each retina. To evaluate the density of vascular cells in the capillaries, the mean number of EC or PC was recorded.

### Visualization of retina vasculature with quantification of avascular area

Vessel obliteration and the retinal vascular pattern were analyzed using retinal wholemounts stained with anti-collagen IV antibody as described previously [17]. The enucleated eyes were fixed in 4% paraformaldehyde for 5 hours then placed in 70% methanol for at least 24 h at -20°C. Retinas were dissected in PBS and then washed and incubated in a blocking buffer (50% fetal calf serum, 20% normal goat serum in PBS) for 2 h. The retinas were incubated with anti-collagen IV (diluted 1:250 in PBS containing 20% fetal calf serum, 20% normal goat serum) overnight followed by a wash with PBS and incubation with secondary antibody CY3 goat-anti-rabbit (Jackson ImmunoResearch; 1:500 dilution prepared in PBS containing 20% FCS, 20% NGS) for at room temperature for 2 h. In some cases, retinas were co-stained with Isolectin B4 to aid identification of the area of capillary loss. Retinas were mounted and viewed by fluorescence microscopy. Images were captured in digital format using a Zeiss microscope (Carl Zeiss, Chester, VA). The central area of capillary loss was quantified, as a percentage of the whole retina area, from the digital images in masked fashion using Axiovision software (Carl Zeiss, Chester, VA). Quantification of vitreous neovascularization was performed as previously described by Stahl et al. [18, 19].

### Imaging of the hyaloid vasculature

Hyaloid vessels from 10 week old mice were imaged using a Micron III indirect camera (*Phoenix Research Labs*). Mice were anesthetized using ketamine/Xylazine and atropine was used to dilate eyes. Fundus images were taken prior to an intraperitoneal injection of 50 μl sodium fluorescein (10%; *Altaire Pharmaceuticals*) while the retina was in focus on the Micron III this was followed by imaging the hyaloid vessels filled with fluorescein.

To visualize the hyaloid vessels in P10 mice, the eyes were enucleated and briefly fixed in 4% paraformaldehyde and stored in methanol at -20°C until dissection. The eyes were then rehydrated in phosphate buffered saline (PBS). The sclera, choroid and retinal pigment epithelial layers were separated from the retina while the lens was still in the vitreous. The lens was then extracted gently from the vitreous using fine forceps. The retina was transferred to a clean cell culture dish (without PBS) and a pre-warmed 6% gelatin solution was injected and allowed to solidify on ice for 2–3 minutes. To prevent the retina from drying out, cold PBS was added to the dish and left on ice for one hour. Gelatin protected hyaloid vessels were removed from the eye cup in cold PBS and under the dissection microscope and placed on a glass slide. Warm PBS was used to dissolve and wash out the gelatin and the hyaloid vessels orientated using a fine forceps. The preparations were allowed to air dry overnight, DAPI stained and imaged by fluorescent microscopy.

### Apoptosis and proliferation

Eyes were enucleated, fixed for 3 minutes in 4% paraformaldehyde and stored in methanol at -20°C overnight. The dissected retinas were placed in PBS for 30 minutes, fixed in 3% paraformaldehyde for 30 minutes and washed three times in PBS. The retinas were then transferred to new tubes, rinsed and blocked in 50% blocking buffer (see above) for 24 hours at room temperature. Next the retinas were incubated with anti-cleaved caspase-3 (1:50; Cell Signaling clone D3E9 # 9579) or Ki-67 (1:50 Cell Signaling clone D3B5 #12075) in 2.5% BSA, 0.4% Triton X-100 and 5% blocking buffer for 24 hours at 4°C while rocking, washed 5 times in PBS, one time in 2.5% BSA, 5% blocking buffer and 0.4% Triton X-100 (20 minutes at room temperature) and then rocked in 50% blocking buffer at room temperature for 30 minutes. The retinas were then incubated with the appropriate secondary antibody (1:500; Jackson ImmunoResearch Laboratories) in 2.5% BSA, 5% blocking buffer and 0.4% Triton X-100 rocking for 2 hours at room temperature. The samples were washed three times in PBS for 10 minutes and fixed in 3% paraformaldehyde for 30 minutes at room temperature. The retinas were then washed 3 times in PBS, transferred to new tubes and washed once more in PBS. The retinas were then incubated in Isolectin B4-FITC (1:100: Vector Labs) for 90 minutes and washed. The samples were mounted in mounting medium with DAPI (Southern Biotech). For quantification, the numbers of cleaved caspase-3 or Ki-67 positive cells on the blood vessels were determined per retina.

### qPCR analysis

Two mouse retinas were homogenized in 1 mL of Trizol (Invitrogen). First, 0.2 mL of chloroform was added to each sample, followed by vortexing for 20 sec, and incubated at room temperature for 2–3 min. Samples were then centrifuged at 10,000xg for 18 min at 4°C and the aqueous phase transferred to a new tube. An equal volume of RNA-free ethanol was added to each sample. Samples were loaded onto an RNeasy column (Qiagen kit) then centrifuged for 30 sec at 8,000xg. The flow-through was discarded and 700 μL of Buffer RW1 added to the column and centrifuged for 30 sec at 8,000xg. Samples were washed twice with 500 μL of Buffer RPE. The columns were transferred to a new 1.5 mL collection tube and 40 μl of RNase-free water was added directly onto the column membrane and incubated for 1–2 min at room temperature. RNA was eluted by centrifuging for 1 min at 8,000xg.

Complimentary deoxyribonucleic acid (DNA) synthesis was performed from 1 μg of total RNA using Sprint^™^ RT Complete-Double PrePrimed kit (Clontech; Mountain View, California) according to the manual. For quantitative polymerase chain reaction analysis, 1 μl of each cDNA (dilution 1:10) was used as template in qPCR assays, performed in triplicates on Mastercycler Realplex (Eppendorf; Hauppauge, NY) using the SYBR qPCR Premix (Clontech) with specific primers;

Bim-F (AGTGTGACAGAGAAGGTGGACAATT)

Bim-EL-R (GGGATTACCTTGCGGTTCTGT)

Bim-L-R (GCTCCTGTCTTGCGGTTCTG)

Bim-S-R (GTATGGAAGCTTGCGGTTCTGT)

RpL13A-F (TCTCAAGGTTGTT CGGCTGAA)

RpL13A-R (GCCAGACGCCCCAG GTA)

Amplification parameters were as follows: 95°C for 2 min; 40 cycles of amplification (95°C for 15 sec, 60°C for 40 sec); dissociation curve step (95°C for 15 sec, 60°C for 15 sec, 95°C for 15 sec). Standard curves were generated from known quantities for each target gene of linearized plasmid DNA. Ten times dilution series were used for each known targets, which were amplified using SYBR-Green qPCR. The linear regression line for ng of DNA was determined from relative fluorescent units (RFU) at a threshold fluorescence value (Ct) to gene targets from retina extracts and normalized by the simultaneous amplification of RpL13A (a housekeeping gene) for all samples. Mean and standard deviation of all experiments performed were calculated after normalization to RpL13A.

### Analysis of formation of the deep vasculature

Eyes from P10 mice were enucleated and fixed for 3 minutes in 4% paraformaldehyde and stored in methanol at -20°C overnight. The retinas were dissected and placed in PBS for 30 minutes, fixed in 3% paraformaldehyde for 30 minutes and washed three times in PBS. Retinas were then wholemount stained with Isolectin B4 as described above (proliferation and apoptosis) and mounted. For quantification, the mean number of vertical sprouts were determined per retina. The central area of vertical sprouting was quantified from the digital images in masked fashion using ImageJ (Carl Zeiss, Chester, VA).

### Statistical analysis

Statistical differences between groups were evaluated with ANOVA with Tukey’s Multiple Comparison Test. Mean ± standard deviation is shown. Comparison between wild-type and Bim^EC^ or wild-type and Bim^PC^ was confirmed with a T-test. qPCR data was analyzed with a One-Way ANOVA with subgroup p-values adjusted using Dunnett's multiple comparisons test.

## Results

### Attenuation of hyaloid vessel regression in Bim^EC^ or Bim^PC^ mice

The immature lens, retina and vitreous are nourished by the pupillary membrane (PM) and hyaloid vessels-hyaloid arteries, tunica vasculosa lentis (TVL), and vasa hyaloidea propria (VHP) [20]. Regression of the hyaloid vasculature is an apoptosis-driven process. Failure of the hyaloid vasculature to regress, termed persistent fetal vasculature or persistent hyperplastic primary vitreous, is one of the most common congenital malformation syndromes of the eye [21]. In the mouse, hyaloid vessel regression begins prior to weaning, with complete regression by six weeks of age [6].

**Fig 2:**
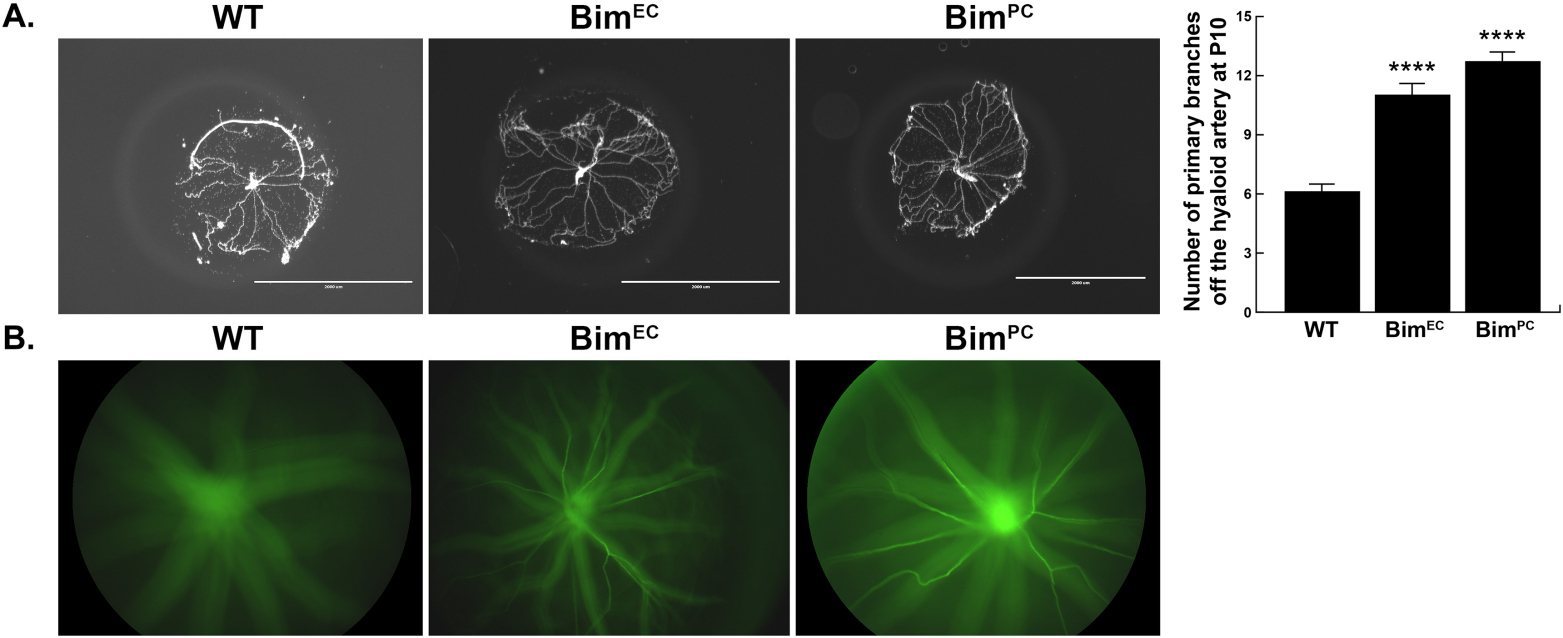
Hyaloid regression is regulated by Bim expression in vascular cells. **Panel A** demonstrates DAPI stained hyaloid vessel preparations from postnatal day 10 wild-type and conditional Bim knockout mice. Please note lack of Bim expression in EC or PC attenuated hyaloid vessel regression. (n =, ****P<0.0001) Scale bar equals 2000 μm. In **Panel B**, a representative image is shown of hyaloid vessels from 10 week old mice imaged using a Micron III indirect camera. Fundus images were taken prior to an intraperitoneal injection of 50 μl sodium fluorescein. While the retina was in focus on the Micron III, images were taken as the hyaloid vessels filled with fluorescein. These studies were performed by imaging hyaloid vessels from 5 mice (n = 5) with similar results.

Although Bim has been shown to be expressed in hyaloid EC and PC [22], Bim-driven apoptosis of hyaloid EC or PC during hyaloid vessel regression is not completely understood. Our previous studies demonstrated attenuated hyaloid vessel regression in global Bim -/- mice [6]. Here we assessed the necessity of Bim expression in EC or PC during hyaloid vessel regression by generating mice homozygous for the Bim^Flox/Flox^ allele which also expressed VE-cadherin-cre (Bim^EC^) or Pdgfrb-cre (Bim^PC^). We observed approximately 2-fold more primary branches off the hyaloid artery in P10 Bim^EC^ or Bim^PC^ mice compared to their wild-type counterparts (Fig 2A). Next, Micron III imaging was used to determine whether the hyaloid vasculature persisted into adulthood in Bim conditional mice. As anticipated, complete regression of the hyaloid vasculature was observed in 10 week old wild-type mice. However, hyaloid vessels were still evident in 10 week old Bim^EC^ and Bim^PC^ mice (Fig 2B). Thus, persistence of hyaloid vessels in Bim^EC^ and Bim^PC^ mice suggests that Bim expression in EC and/or PC is needed for complete regression of the hyaloid vasculature.

### Bim^EC^ mice demonstrated enhanced formation of the deep vasculature

Formation of the rodent retinal vasculature is the result of the finely orchestrated movement of retinal vascular cells including EC and PC. During the first week of life, a superficial layer of vessels initiates from the area around the optic disc spreading radially toward the retinal periphery. During the second and third week of life, these vessels sprout deep into the retina and spread perpendicularly to the superficial layer forming the deep and intermediate retinal vessels. By the third week, the retina is completely vascularized but vascular remodeling and pruning continue for three more weeks [17, 23, 24].

**Fig 3:**
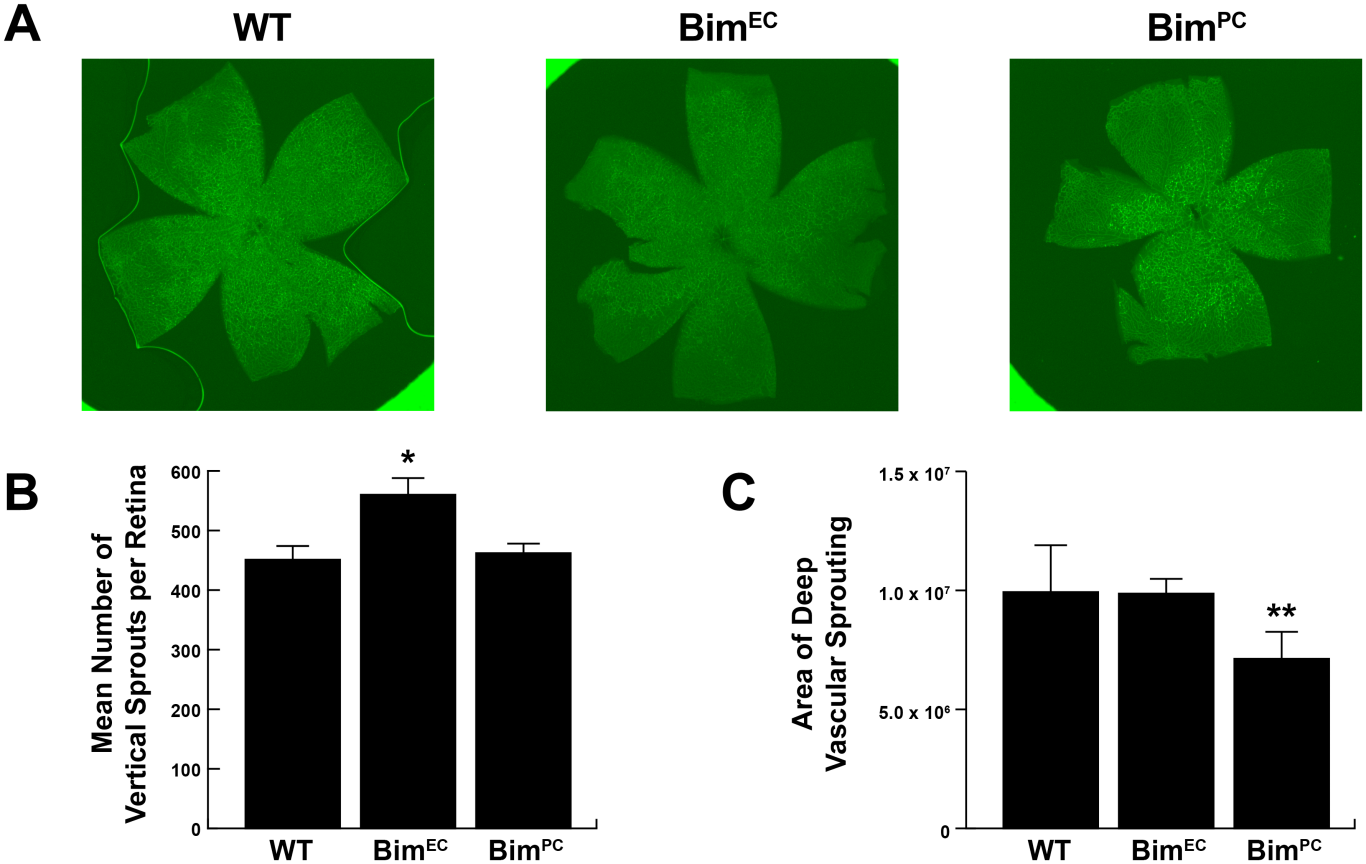
Enhanced deep vascular plexus formation in Bim^EC^ mice. In **Panel A**, retinas from P10 wild-type, Bim^EC^ and Bim^PC^ mice were wholemount stained with Isolectin B4 and the superficial layer imaged (25x). In **Panel B** the mean number of vertical sprouts were quantified in each retina. (n = 5, *P<0.05). In **Panel C**, the area covered by the vertical sprouts for each retina was quantified. (n = 5, **P<0.01)

Our previous studies in global Bim -/- mice demonstrated enhanced formation of the deep vascular plexus [6]. Here we assessed whether lack of Bim expression in EC or PC was sufficient to enhance vertical sprouting into the deep vascular plexus. Retinas from P10 wild-type, Bim^EC^ and Bim^PC^ mice were wholemount stained with isolectin B4 to visualize the retinal vasculature. Fig 3 demonstrates enhanced vertical sprouting in Bim^EC^ mice compared to their wild-type counterparts. Although Bim^PC^ mice had fewer sprouts per retina, the sprouting area was also smaller than wild-type mice making their sprouting deep into the vascular plexus at a higher density than wild-type mice over the area covered (Fig 3). Therefore, loss of Bim expression enhanced vertical sprouting toward the deep vascular plexus.

### Increased numbers of retinal vascular cells in conditional Bim mice

**Fig 4:**
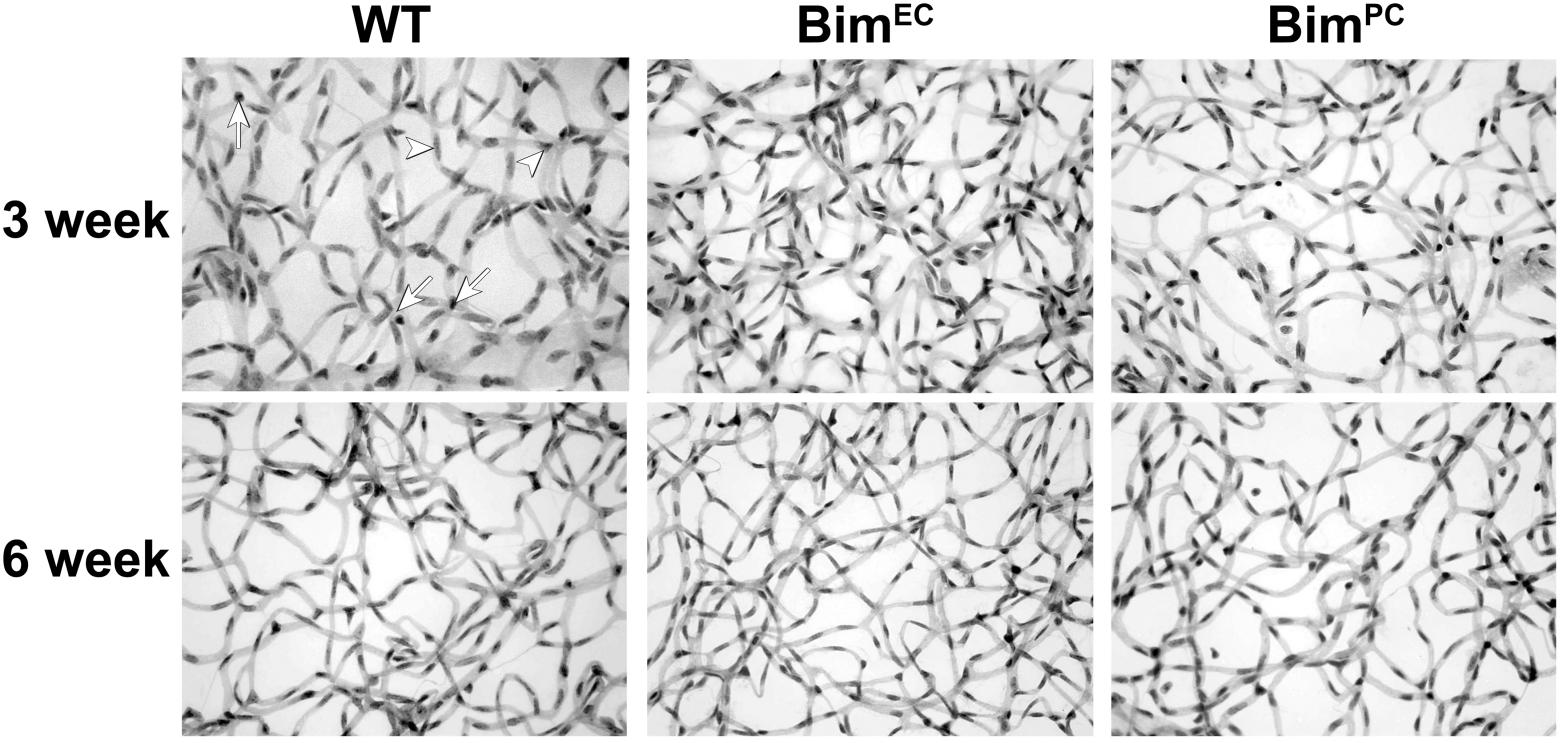
Increased EC number in retinas from conditional Bim mice. Retinas from P21 and P42 mice were prepared by trypsin digest and HE/PAS staining. EC and PC were then quantitated per x400 field of view. Scale bar = 100 μm. Experiments were repeated with eyes from >10 mice with similar results. Four quadrants per eye were counted for the quantitative assessment of this data is summarized in Table 1.

Unnecessary retinal vessels are removed by an apoptosis-driven remodeling, a process which is completed in the mouse by 6 weeks of age. Next, we assessed trypsin digests of retinas from 3-week and 6-week-old wild-type, Bim^EC^ and Bim^PC^ mice to assess retinal EC and PC numbers (Fig 4). Lack of Bim expression in EC (Bim^EC^ mice) prevented EC loss during retinal vascular remodeling (Table 1). Bim^PC^ mice demonstrated increased PC numbers at 3 and 6 weeks of age compared to wild-type mice (Table 1). Bim^PC^ mice also demonstrated increased retinal EC numbers compared to their wild-type counterparts at 6 weeks of age. Therefore, Bim expression in EC and/or PC aids postnatal retinal vascular developmental remodeling.

**Table 1:** Retinal vascular cell numbers.

	Age	Control	Bim^EC^	Bim^PC^
**Pericytes (PC)**	**P21**	**28.35 ± .56**	**28.0 ± .77**	50.93 ± 1.21****
**Endothelial Cell (EC)**	**P21**	**187.5 ± 4.31**	200.0 ± 2.94*	**187.1 ± 5.59**
**Pericytes (PC)**	**P42**	**25.83 ± .63**	22.17 ± .63***	45.00 ± 1.47****
**Endothelial Cell (EC)**	**P42**	**157.2 ± 3.17**	195.6 ± 3.37*****	179.3 ± 4.86***

Number of Cells Per High Power Field at (x400). The P values were calculated by comparing samples from Control to Bim^EC^ or Bim^PC^ mice at the ages noted:

*P<0.05;

**P<0.01;

***P<0.001;

****P<0.0001; and

*****P<0.00001.

### Decreased proliferation and apoptosis in retina from mice conditionally lacking Bim expression

**Fig 5:**
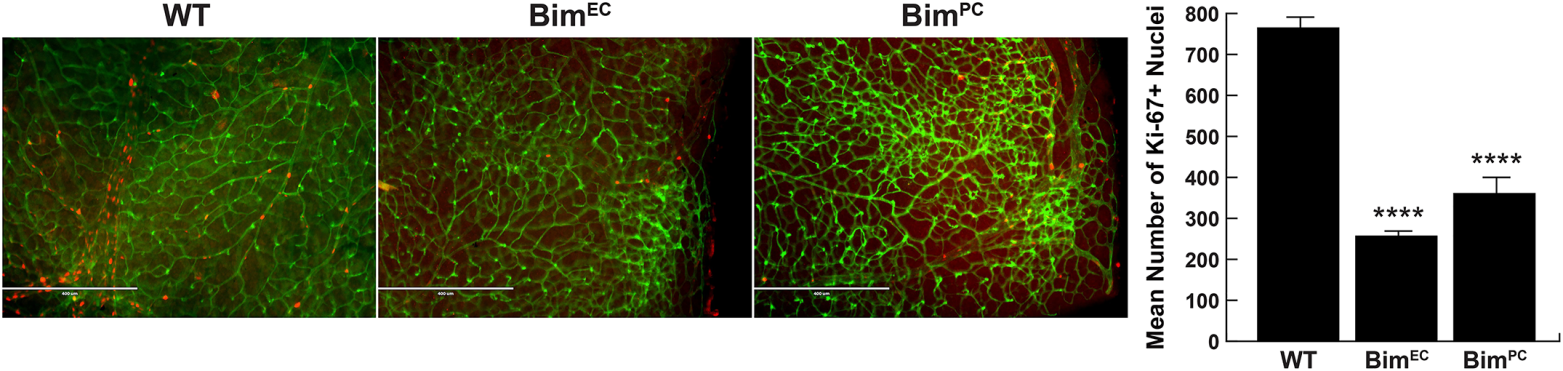
Decreased proliferation in retinas from conditional Bim mice. Proliferating cells in P14 wild-type, Bim^EC^ and Bim^PC^ mouse retinas were assessed by anti-Ki-67 staining (red) and co-stained with Isolectin-B4-FITC (green) to visualize the vasculature. The data in each bar are the mean number of Ki-67 positive cells counted in each retina. Please note that the number of proliferating cells is lower in retinas from conditional Bim mice compared to their wild-type counterparts (n = 6, ****P< 0.0001). Scale bar equals 400 μm.

**Fig 6:**
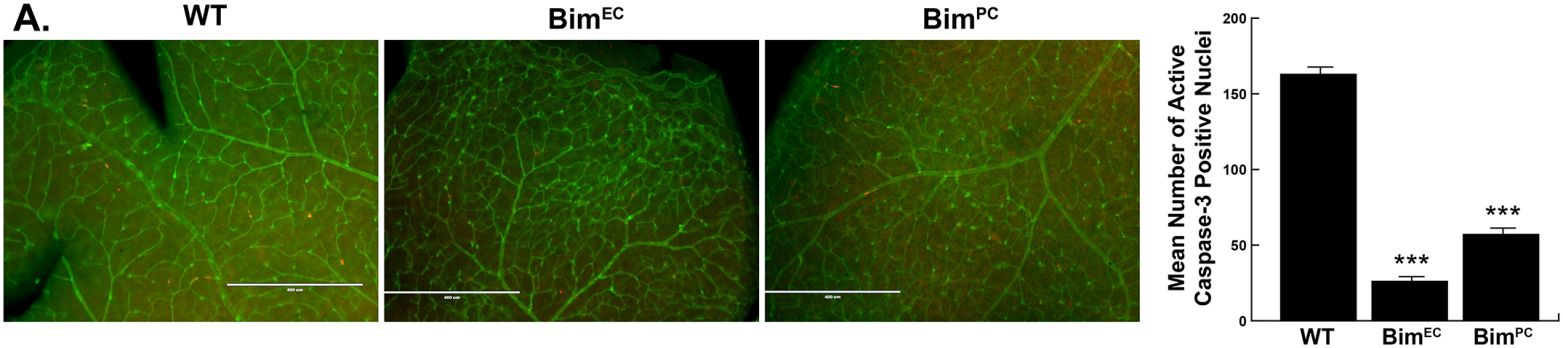
Decreased apoptosis in retinas from conditional Bim mice. Retinas from P14 wild-type, Bim^EC^ and Bim^PC^ mice were wholemount stained with anti-cleaved caspase 3 (red) and co-stained with Isolectin-B4-FITC (green). The intermediate layer was imaged and the number of cleaved caspase 3 positive cells were quantified. The data in each bar are the mean number of cleaved caspase 3 positive cells counted in the vasculature of each retina. (n = 6, ***P<0.001) The scale bar equals 400 μm.

Unbalanced proliferation and apoptosis can affect retinal vascular density by impacting retinal EC and PC numbers. Retinal vascular cell proliferation reaches its maximal level at P14 and declines significantly by P21 [25]. Since conditional Bim mice demonstrated increased numbers of retinal EC and/or PC, we next assessed whether proliferation and apoptosis levels were affected. Both proliferation and apoptosis were significantly decreased in mice conditionally lacking Bim, albeit at different rates. Retinas from Bim^EC^ mice displayed a three-fold decrease in proliferation of vascular associated cells while Bim^PC^ mice had a two-fold decrease of proliferation of vascular associated cells compared to their wild-type counterparts (Fig 5). We also observed decreased numbers of cleaved caspase-3 positive cells in conditional Bim mice. Bim^EC^ mice displayed a six-fold decrease and Bim^PC^ a three-fold decrease in the number of apoptotic cells compared to their wild-type counterparts (Fig 6). These results are consistent with decreased apoptosis resulting in increased numbers of EC and/or PC.

### Wild-type, Bim^EC^ and Bim^PC^ mice exhibited similar hyperoxia-mediated retinal vessel obliteration and neovascularization during OIR

**Fig 7:**
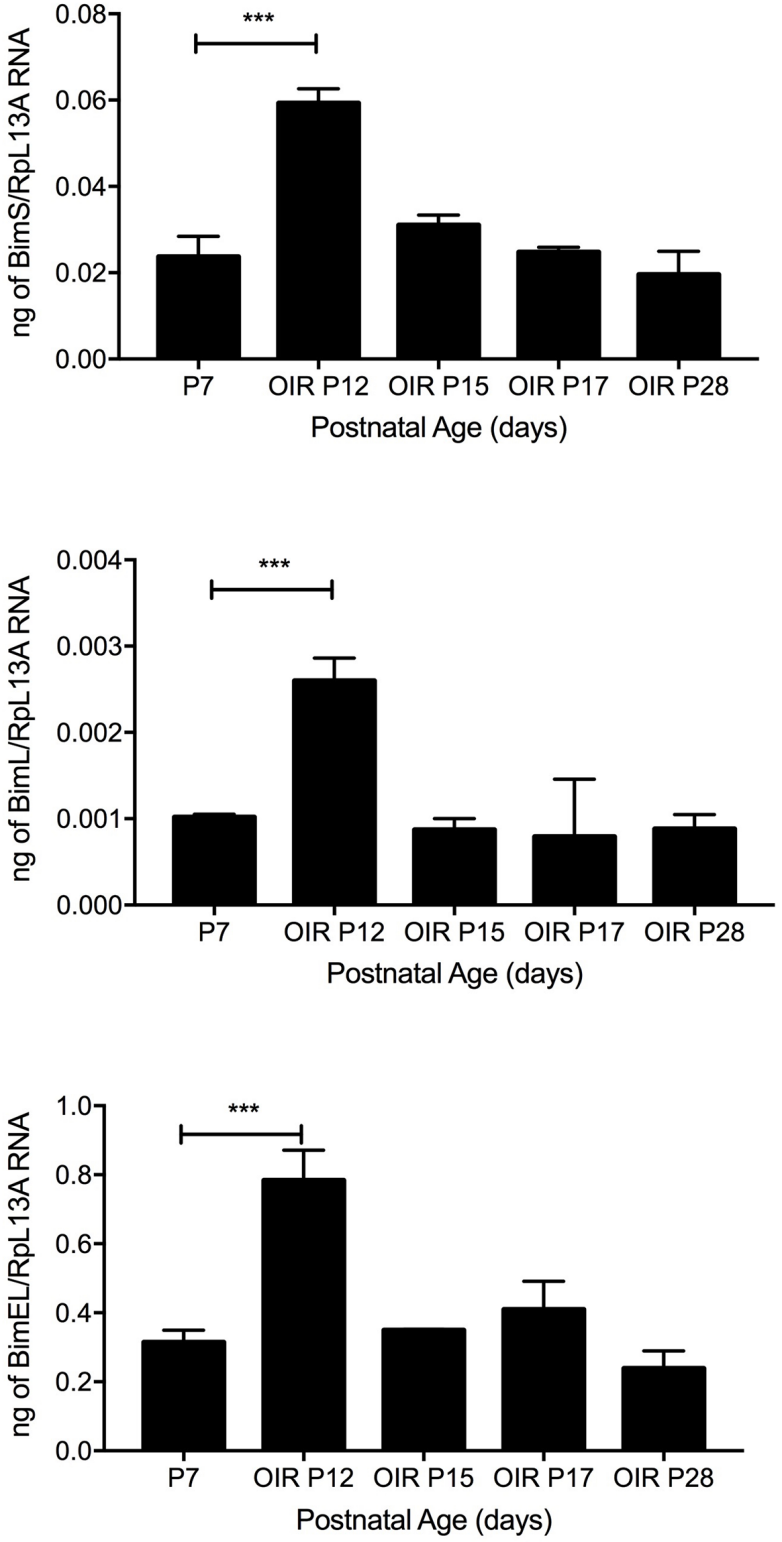
Bim expression increases during hyperoxia. Wild-type mice were subjected to OIR at P7. RNA was prepared from the retina of wild-type mice at the noted times. Bim expression was analyzed by qPCR. Please note increased Bim expression with hyperoxia (P12). Samples were done in triplicate and repeated twice. (n = 6, ***P<0.001).

The developing retinal vasculature is sensitive to changes in oxygen level which predisposes the retina to OIR. Here, P7 mice were subjected to hyperoxia for 5 days and then returned to room air for up to 5 days [7]. The exposure of developing retinal vasculature to high oxygen during the first phase prevents growth of additional vessels and promotes the loss of existing vessels due to diminished expression of VEGF (phase I). When these mice are returned to room air, the retina becomes ischemic and promotes growth of new blood vessels (phase II), which grow into the vitreous. Bim isoform expression was assessed in retinas from wild-type mice harvested at P7 (prior to hyperoxia), P12 (upon return to room air following hyperoxia), P15 (hyperoxia followed by 3 days room air), P17 (hyperoxia followed by 5 days room air) and P28 (hyperoxia followed by 16 days room air). Expression of BimEL, BimL and BimS increased with hyperoxia (P12) and subsequently decreased upon exposure of the mice to room air (Fig 7). Therefore, increased Bim isoform expression during hyperoxia occurred at time when retinal vessel obliteration has been observed in wild-type mice [6, 7].

**Fig 8:**
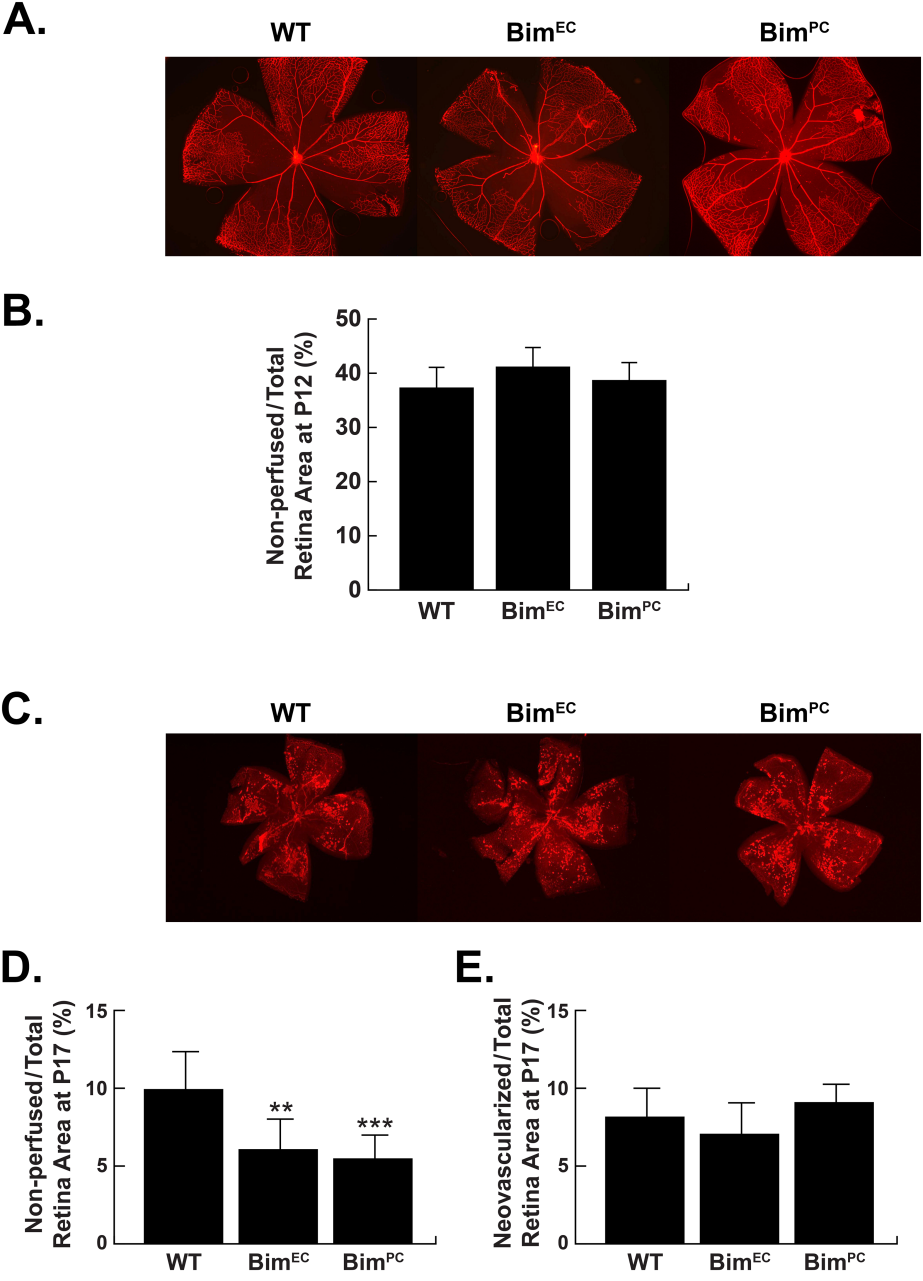
Lack of EC or PC Bim expression does not attenuate hyperoxia-driven vessel obliteration. Quantitative assessment of vessel obliteration and neovascularization in mice previously exposed to a cycle of hyperoxia and room air (OIR). Retinas from P12 and P17 Bim^Flox/Flox^ (WT) and conditional Bim littermates were wholemount stained with anti-collagen IV to visualize the vasculature (Panels A,C). The area of vessel obliteration relative to the whole retina was quantitated at P12 (n = 5, P>0.05, Panel B) and the non-perfused area remaining relative to the whole retina at P17 was quantitated (Panel D; n = 5, **P<0.01, ***P<0.001). Panel E is a quantitative assessment of the neovascularization (n = 5, P> 0.05). For Panel A magnification is at 2.5 X while Panel C is at 1.25 X.

We previously demonstrated that mice globally lacking Bim expression were protected from hyperoxia-mediated vessel obliteration and ischemia-mediated neovascularization during OIR [6]. Here, we determined whether lack of Bim expression in retinal EC or PC could protect the retinal vasculature from hyperoxia-mediated retinal vessel obliteration and subsequent neovascularization. In contrast to what we had previously observed in global Bim deficient mice following OIR, P17 wild-type, Bim^EC^ and Bim^PC^ mice demonstrated similar amounts of neovascularization (Fig 8E), with similar areas of vessel obliteration surrounding the optic nerve at P12 (Fig 8A and 8B). However, the non-perfused area at P17 in Bim^EC^ and Bim^PC^ mice was approximately 50% less than their control littermates (Fig 8D). Thus, loss of Bim expression in EC or PC alone was not sufficient to prevent hyperoxia mediated vessel obliteration and ischemia-mediated neovascularization.

## Discussion

Dysregulation of pro-apoptotic Bcl-2 family members prevents hyaloid vessel regression [6, 22, 26]. Although Bim is expressed in hyaloid EC, PC and ocular macrophages [22], its function in these cells during hyaloid regression is poorly understood. The transient hyaloid vascular system begins to regress with the development of the retinal vasculature [27]. The regression of the hyaloid artery being accompanied by increased PC apoptosis [28, 29]. The VHP, which is closest to the retina, is the last to regress. Here we show persistence of the hyaloid vasculature in Bim^EC^ and Bim^PC^ mice suggesting that hyaloid vascular cells must be susceptible to apoptosis for complete regression to occur. Although macrophages have been proposed to play an essential role in hyaloid vessel regression [28, 30, 31], lack of neuronal VEGFR2 expression facilitated persistence of hyaloid vessels due to decreased EC apoptosis even though macrophage numbers were unchanged [32]. This phenotype was normalized by neuronal deletion of VEGF [33]. Therefore, for macrophages to facilitate hyaloid vessel regression, EC and/or PC must be capable of undergoing apoptosis.

Retinal vascular remodeling removes excess capillaries in response to changing oxygen requirements during development, resulting in a more streamlined and stable network to effectively maintain tissue oxygenation. In contrast, during pathological states such as retinopathy of prematurity (ROP), vascular segment drop out during hyperoxia destabilizes the retinal vascular network and disrupts tissue oxygenation upon return to room air. Although retinal vascular remodeling is an apoptosis-driven process regulated in part by Bim and Bcl-2 [6, 34], it remains to be determined whether Bim-driven apoptosis of retinal PC or EC facilitates developmental or pathological retinal vascular remodeling. In recent years PC have been shown to be important regulators of not only vascular development but also stabilization, maturation and remodeling of the vasculature [35]. PC afford protection to retinal EC and therefore these cells are interdependent for the maintenance of competent microvessel function. When PC lacked Bim expression, we observed both an increase in PC and EC numbers compared to their wild-type counterparts at 6-weeks of age. In contrast, lack of EC Bim expression resulted only in increased EC numbers in the retinal vasculature. When Bim expression was lacking in either EC or PC, EC remodeling decreased. These data are consistent with PC protecting retinal EC during remodeling. Thus, delineating how modulation of Bim expression impacts retinal EC and PC apoptosis will give us further insight into the interactions between these cell types and their contribution to vessel rarefaction during various disease states.

ROP is a vision-threatening disease due to hyperoxia-driven vascular apoptotic regression and subsequent ischemia-driven retinal neovascularization [36]. During hyperoxia attenuation of retinal vascular development and loss of existing blood vessels (phase I; an apoptosis driven process) is observed. When preterm infants are hypoxemic their ischemic retina initiates a proangiogenic response, including increased VEGF production which results in pathological growth of new blood vessels (phase II). We recently showed that lack of Bim expression increased VEGF expression in retinal vascular cells [2, 8, 9] and protected the developing retinal vasculature from hyperoxia-mediated vessel obliteration and subsequent ischemia-mediated neovascularization during OIR (phase I and II) [6]. These global Bim deficient mice demonstrated only about a 1% loss of retinal vessels following hyperoxia and little to no neovascularization compared to control mice [6]. Here we show that Bim^EC^ or Bim^PC^ mice had an area of hyperoxia-mediated vessel obliteration at P12 and subsequent ischemia-driven neovascularization similar to control littermates. However, by P17 following OIR, the non-perfused area was approximately 2-fold less in mice conditionally lacking Bim in EC or PC suggesting revascularization was enhanced in the absence of Bim. Perhaps this is not surprising given we previously demonstrated increased migration of Bim -/- EC and PC compared to wild-type cells [3], which may be advantageous during revascularization. Together these studies indicate that simply inhibiting Bim expression in a single vascular cell type will not suffice since near complete inhibition of hyperoxia-mediated vessel obliteration appears to be required to prevent ischemia-driven neovascularization. This notion is consistent with our previous studies in TSP1 deficient mice where hyperoxia-mediated vessel loss was decreased about 2-fold while the level of neovascularization was similar to control mice [17]. Thus, hyperoxia-mediated vessel obliteration (phase I) may be influenced by Bim expression in other ocular cell types including neuronal and/or glial cells or multiple vascular cell types.

In summary, our data indicates that EC and PC Bim expression modulates developmental retinal vascular remodeling. Expression of Bim in other cellular components of the retina may make significant contributions to hyperoxia-mediated vessel regression. Therefore, modulation of Bim expression may provide a suitable target to promote or inhibit vessel regression.
